# The design and evaluation of a health education control for comparison with cognitive behavioural therapy for individuals with acquired brain injury

**DOI:** 10.1186/s40814-022-01070-8

**Published:** 2022-06-06

**Authors:** Lucy Ymer, Adam McKay, Dana Wong, Jennie Ponsford

**Affiliations:** 1 School of Psychological Sciences, Turner Institute for Brain and Mental Health, Monash University, Melbourne, VIC Australia; 2 Monash Epworth Rehabilitation Research Centre, Melbourne, VIC 3121 Australia; 3 Department of Psychology, Epworth Rehabilitation, Melbourne, VIC Australia; 4 School of Psychology and Public Health, La Trobe University, Melbourne, VIC Australia

**Keywords:** Health education, Control condition, Cognitive behavioural therapy, Acquired brain injury, Sleep, Fatigue

## Abstract

**Background:**

In psychological research, control conditions in the form of “treatment as usual” provide support for intervention efficacy, but do not allow the attribution of positive outcomes to the unique components of the treatment itself. Attentionally and structurally equivalent active control conditions, such as health education (HE), have been implemented in recent trials of cognitive behavioural therapy (CBT). However, descriptions and evaluations of these control conditions are limited. The aims of this paper were to (i) provide a detailed description and rationale for a novel HE active control condition and (ii) to evaluate the face validity, treatment integrity and feasibility of HE.

**Method:**

We developed a HE active control similar in structure and duration to a CBT intervention for reducing sleep disturbance and fatigue (CBT-SF) in a pilot randomised controlled trial (*n* = 51). Face validity was measured using post-treatment participant satisfaction and helpfulness ratings for fatigue and sleep symptoms, treatment fidelity was measured with integrity monitoring ratings from an independent expert and feasibility was measured with completion and attrition rates. HE and CBT-SF groups were compared using Wilcoxon rank-sum tests and chi-square tests of independence.

**Results:**

There were no significant differences in participant ratings of overall satisfaction between HE (*n* = 17) and CBT (*n* = 34) or in how helpful each intervention was for fatigue symptoms. Participants rated helpfulness for sleep symptoms higher in the CBT-SF group compared to HE. Integrity monitoring ratings were not significantly different for overall treatment delivery and therapist competency, but HE had greater module adherence than CBT-SF. There were no significant differences in completion or attrition rates between groups.

**Conclusion:**

Our findings suggest that the HE control had adequate face validity, was delivered with fidelity and was feasible and suitable for use as a comparator for CBT-SF. In providing a real-world example of practical and theoretical issues we considered when designing this control condition, we aim to provide a framework and guidance for future investigators.

**Trial registration:**

ACTRN12617000879369 (registered 15/06/2017) and ACTRN12617000878370 (registered 15/06/2017).

## Background

Randomised controlled trials (RCT) are critical in providing evidence in the evaluation of a range of treatments and interventions. In an RCT, the efficacy of a treatment is determined relative to the outcome of the control group, and therefore, the careful selection and design of control conditions is vital in protecting the internal validity and statistical power of a trial [1, 2]. A common choice for control conditions in pharmacological research is placebo medication; however, the protocol is not so well-defined in trials of psychological interventions. Although many psychological treatments have demonstrated positive improvements in symptoms when evaluated on their own, a well-selected control condition will allow attribution of the outcomes to the specific treatment itself [1].

The most common control conditions used in RCTs for psychological interventions are specific treatment controls, non-specific controls (also known as active controls or attentional controls), treatment as usual (TAU) and no treatment or waitlist [1, 3]. Each design has its own advantages and disadvantages, and selection depends on the primary aims and purpose of the RCT [2]. Of the most relevance to the current paper are non-specific active controls. A non-specific active control condition aims to account for the non-specific factors (or “common factors”) of engaging in a psychological intervention [1, 2]. This allows the attribution of improved outcomes to the unique elements of the comparison treatment, such as specific techniques, strategies or skills learned in session [4, 5]. The use of a non-specific active control over a less active control group (such as TAU) is also likely to increase recruitment and retention rates [4].

The content and design of non-specific active control conditions in psychological interventions vary between RCTs and heavily depend on the comparison intervention. Despite this, there is limited research that details the selection, design and implementation of non-specific control conditions and the rationale behind their construction. To effectively implement a non-specific control, Mohr and Spring [1] highlight the importance of manualising to the same degree as the experimental group and subjecting control conditions to the same fidelity monitoring procedures. Additionally, it is important for non-specific controls to not only be defined by prohibitions of certain intervention components (e.g. cognitive or behavioural techniques) but also provide some form of therapeutic intervention that is non-specific in nature [1]. To the author’s knowledge, only one trial to date has quantitatively evaluated a health education control condition on attentional and engagement variables, compared to an active treatment [6]. Raina and Morse [6] assessed the feasibility of administering a cognitive behavioural intervention for fatigue after traumatic brain injury (TBI) compared to a health education control. They compared intervention length, session duration, engagement ratings and homework completion between the groups, finding that the groups were similar on all measures excluding session duration [6]. They did not, however, assess fidelity ratings or any participant variables. Rethorst and Greer [4] collected session checklists and progress notes for their novel health education intervention for exercise, but did not present a formal analysis of engagement or associated outcomes. Lastly, Safer and Hugo [7] evaluated a non-specific psychotherapy control in comparison with dialectical behaviour therapy for binge eating disorder, by defining and measuring six relevant “common factors” of psychotherapy. They compared these common factors using *t*-tests, finding no significant differences between control and experimental groups, other than therapist optimism about patient outcomes [7]. Overall, it is clear that further evidence is required evaluating the utility and rigour of non-specific control conditions.

We recently developed a health education intervention to be used as an active control in a pilot RCT investigating the preliminary efficacy of cognitive behavioural therapy (CBT) for sleep and fatigue (CBT-SF) in individuals with acquired brain injury (ABI) [8]. The preliminary efficacy of CBT-SF in ABI has been demonstrated in pilot studies by Nguyen and Wong [9, 10] when compared to TAU. Our active control pilot RCT, described in more detail below, aimed to build on these findings by comparing the preliminary efficacy of CBT-SF with that of a health education (HE) active control, to account for non-specific effects of engaging in therapy [8]. The first aim of this paper was to provide a detailed description and rationale for the HE design, given that few studies provide sufficient information that can be used to support future developments of control conditions. A second aim was to evaluate the face validity, treatment integrity and feasibility of the HE condition compared with an active treatment (CBT-SF). It was hypothesised that there would be no significant differences in face validity (participant ratings of overall satisfaction, helpfulness for fatigue and helpfulness for sleep), integrity monitoring ratings (module adherence, therapist competency and overall delivery) and feasibility (completion rates and attrition rates) between HE and CBT-SF.

## Method

### Rationale for health education as a control condition

In selecting the topic for our active control, we considered the use of HE or sleep hygiene. As described above, HE has been established as an ineffective, yet attentionally equivalent control condition in studies of exercise [4, 11], and as a feasible control group in various trials investigating psychological interventions such as CBT for insomnia, anxiety, depression and quality of life in non-ABI populations [12–15]. To our knowledge, only two studies have used HE as a control condition in a sample of individuals with TBI [6, 16]. At the time of the HE design process, only the study by Raina and Morse et al. [6] was available to use as a reference.

Sleep hygiene interventions have been used in past studies as a comparator to CBT for insomnia and were therefore considered as a potential control condition for our trial [17]. Sleep hygiene focuses on four key areas: sleep homeostatic factors, circadian factors, medication and drug effects and arousal in sleep setting [18]. A recent systematic review and meta-analysis found sleep hygiene to have small to moderate effects in improving symptoms of insomnia, despite remaining less effective than CBT and mindfulness-based sleep interventions [17]. Recent evidence also suggests that when used in isolation or in combination with other treatments, sleep hygiene may reduce sleep difficulties in individuals with TBI [18]. We deemed that sleep hygiene, while potentially having greater face validity than HE for treating sleep and fatigue difficulties, was more likely to result in positive improvement in symptoms and was therefore less appropriate to use as a control condition in the context of ABI. In addition, elements of sleep hygiene were included as behavioural techniques in our CBT-SF design. In our aim to select an intervention to control for attentional and therapeutic effects while avoiding active components of CBT-SF, we decided to use HE.

### Design of a health education control

#### Identifying and accounting for non-specific factors of psychotherapy

The first step in conceptualising and designing our HE control condition was to consider which non-specific factors we wished to control for in our design [7]. These non-specific factors, or “common factors” of psychotherapy, play an important role in the mechanism of therapeutic change, alongside the specific components of the treatment itself [19]. The aim of our HE control condition was to hold constant the overall treatment focus and the amount of attention and treatment contact with both researchers and treating clinicians.

In an attempt to ensure that these common factors were accounted for by the HE control group, we designed the control condition to be structurally equivalent to the CBT-SF. This included having equal attentional contact with therapists and researchers and matched data collection requirements. Both interventions involved eight 60-min sessions across eight weeks, with one of six clinical neuropsychologists independent of the study team, each of whom provided both HE and CBT-SF interventions. Treatments were manualised, and each therapist received individual training in the application of each intervention. Therapists also received regular supervision. Integrity monitoring was assessed by an experienced clinical psychologist to ensure appropriate adherence to each treatment (described in more detail below).

In session, both treatments provided ample opportunity for the therapist to check in with the participants’ well-being over the past week and discuss any obstacles or challenges they had faced. Therapists were instructed to respond empathically to this general discussion in both conditions. Education was provided in a clear and structured format, although the amount of educational material differed between the groups, with the education component being larger and more detailed in the HE. Therapists delivering HE were instructed to strictly avoid use of any cognitive or behavioural techniques implemented in the CBT-SF. If participants were already using their own strategies or asked about CBT-SF-related techniques, this was neither encouraged nor discouraged by therapists, and conversation was redirected to the relevant HE content.

Lastly, steps were taken to moderate participant expectation and engagement in the HE. This was important given evidence that participants who know they are in a control condition may have poorer outcomes than those who are blinded [20]. To account for this, the study was described to participants as a comparison study, examining the efficacy of HE compared to CBT in treating sleep and fatigue symptoms after ABI, without any implication that one intervention might be better.

#### Identifying the unique elements of the cognitive behavioural therapy intervention

Once we had determined the non-specific factors we wished to account for, the next step was to identify the core components of CBT-SF that we wanted to avoid in the HE control condition [7]. A key set of techniques that we set out to avoid were behavioural sleep interventions, including sleep restriction, stimulus control and sleep hygiene. These have been shown as effective in treating symptoms of insomnia, showing efficacy in only a short amount of time [18, 21, 22]. Behavioural interventions for fatigue such as pacing, activity scheduling, behavioural experiments, activity modification and gradual increases/decreases in activity are also effective in the initial phases of treatment [23, 24]. Our CBT-SF intervention additionally implemented thorough and detailed planning of daily activities, schedules and inclusion of sufficient time to rest and recharge. None of these techniques were set out in the HE control manual, and treatment integrity monitoring was utilised to ensure therapists were not applying or discussing these techniques during HE sessions.

Cognitive restructuring techniques used in CBT for insomnia and fatigue are often beneficial in maintaining long-term gains from treatment. Frequently used cognitive restructuring strategies include cognitive therapy, cognitive refocusing, paradoxical intention, and addressing unhelpful and perpetuating thoughts surrounding symptoms [21, 24, 25]. Our CBT-SF intervention employed these strategies, in addition to relaxation techniques, thought records and time management skills. In the final CBT-SF session, we also planned for setbacks and discussed strategies to maintain gains made during treatment and for relapse prevention. All of these techniques were strictly avoided in the HE, similarly to the behavioural interventions described above.

In addition to the differing content of the two conditions, the application of the therapeutic process in each condition was also of great importance. The CBT-SF aimed to incorporate key components of CBT, particularly the comprehensive facilitation of homework completion. There was detailed planning and problem-solving around homework tasks and an emphasis on homework review each week. This was important given the impact that executive impairments and memory difficulties can have on homework engagement for individuals with ABI and ultimately an individual’s capacity to benefit from CBT [26]. Conversely, the HE control condition provided a predetermined set of information and generalised strategies, and did not include the creation or setting of homework tasks. There was no specific direction from the therapists to implement any strategies at home, nor a detailed review of any changes made over the previous week. Lastly, while both treatments had a manualised set of modules to deliver, the CBT-SF had greater flexibility to be adapted to the individual's presenting set of symptoms while maintaining adherence to the core treatment components, whereas the HE condition did not have this flexibility.

#### Designing the content of the health education active control

After identifying the unique elements of CBT-SF that we wanted to avoid in the HE, the next step was to design the content of the control intervention so that it maintained adequate face validity [7]. Face validity was a key guiding principle in our topic selection process, to ensure equivalent treatment expectations between the two conditions. As stated previously, only two studies to our knowledge have specifically utilised a HE control condition compared to CBT for sleep or fatigue in a TBI population, with only one available at the time of HE design [6, 16]. Other research has implemented HE compared to CBT in alternative populations with sleep and fatigue difficulties, such as cancer [27]. We drew on the brief information provided in these studies, in addition to publications by Rethorst, Greer [4] and Safer and Hugo [7], who described in detail the rationale and design of their active control conditions for their exercise intervention and behavioural group therapy intervention, respectively. Part of our design process also included reviewing publicly available information and seeking clinical expertise from treating neuropsychologists with experience in administering the CBT-SF in the TAU pilot studies [9, 10].

The HE control intervention utilised by Raina and Morse et al. [6] included TBI education, fatigue characteristics, principles of energy conservation (but not directive strategies), healthy eating, exercise and relaxation as the primary topics. Bruggeman-Everts and Wolvers et al. [27] employed fatigue, sleep hygiene, balancing energy during the day and coping with worrying thoughts in their psychoeducational active control for treatment of chronic cancer-related fatigue. In insomnia studies, education topics in active control conditions compared to CBT have included sleep hygiene, education about the specific disease/syndrome, healthy diet, physical activity, vitamins and memory [14, 16, 28]. After review and discussion by the authors, seven topics were chosen for our HE, detailed in Table 1. The final session acted as a summary session, briefly covering all topics that had been discussed during the sessions and closing the treatment.

**Table 1:** Summary of health education active control

Topic	Content
Module 1: TBI/stroke education	Introduction to the intervention TBI/stroke education Common cognitive, behavioural and emotional sequalae that occur following ABI
Module 2: Sleep	Normal sleep, including the regulation of sleep Disturbed sleep following ABI — types, frequency and causes Impact of sleep disturbance on everyday life
Module 3: Fatigue	Definition and types of fatigue Causes of fatigue and associated factors Impact of fatigue on everyday life
Module 4: Exercise and stress	Types of exercise and their benefits Relationship between exercise, sleep and fatigue Summary of stress and how it affects our mind and body Relationship between stress, sleep and fatigue
Module 5: Diet, alcohol and substance use	Healthy diet including foods that improve brain health Relationship between diet, sleep and fatigue Impact of alcohol and substance use on the brain Relationship between alcohol/substance use, sleep and fatigue
Module 6: Cognitive difficulties following ABI	Common cognitive difficulties following ABI, including attention, learning and memory, word finding difficulties and executive functioning Relationship between cognitive difficulties, sleep and fatigue
Module 7: Recovery in ABI	Importance of recovery and rehabilitation after ABI Emotional stages of recovery Factors that affect recovery
Module 8: Summary	Summary of key points from each module Closing therapy sessions

*TBI* Traumatic brain injury, *ABI* Acquired brain injury

### Evaluation of the control intervention in a pilot RCT

#### Sample size

Sample size for the active control pilot RCT was chosen based on large effect sizes obtained on the Pittsburgh Sleep Quality Index (PSQI) in TAU pilot studies [9, 10]. For a pilot trial, Whitehead and Julious [29] suggest a sample size of at least 10 per treatment arm for an anticipated large effect size; however, for high confidence in estimating sample size for the main trial, Sim and Lewis [30] suggest at least 50 participants. Given the expectation of potentially smaller differences between groups than in the TAU pilot study due to the use of an active control, a sample size of approximately 30–50 participants was justified for the active control pilot RCT.

#### Participants

Fifty-one individuals with sleep or fatigue problems following TBI or stroke were randomised at a 2:1 ratio into an 8-week one-on-one CBT-SF programme (*n* = 34), adapted for cognitive impairments, or an 8-week one-on-one HE control condition (*n*= 17) [8]. Participants were identified by community clinicians in response to advertising on email lists, word of mouth, in a longitudinal TBI research database or via self-referral through advertisements on brain injury organisational websites. They completed the study either in person (*n* = 21) or via telehealth (*n* = 30), to provide data for a secondary analysis of treatment delivery mode. Administration processes and materials for all treatment and research appointments were equivalent across in person and telehealth modes. The primary outcome was sleep quality, measured by the PSQI. Secondary outcomes included fatigue, depression, quality of life, daytime sleepiness, objective sleep parameters on actigraphy, self-efficacy and time spent in productive activity. These outcomes were measured at baseline, post-treatment, 8-weeks post-treatment and 16-weeks post-treatment. Post-treatment interviews were also conducted with all participants to evaluate helpfulness for sleep and fatigue symptoms and overall satisfaction with treatment. These are described in more detail below. Recruitment is ongoing, and post-treatment interviews continue to be conducted with participants in both interventions.

### Evaluating the health education active control

#### Face validity

As previously stated, face validity was a key guiding principle in the design of our HE control, and participant satisfaction was critical for evaluating perception of the HE control as a valid treatment for sleep and fatigue [7]. In order to measure and assess this, we conducted post-treatment phone interviews with all participants who completed either CBT-SF or HE. Specifically, we sought feedback relating to overall satisfaction with the treatment and how helpful they found the treatment for reducing their fatigue and sleep symptoms. These questions were rated on a 5-point scale, respectively: 1 = “very dissatisfied”, 2 = “dissatisfied”, 3 = “somewhat satisfied”, 4 = “moderately satisfied” and 5 = “very satisfied” and 1 = “it made things worse”, 2 = “did not really help”, 3 = “helped somewhat”, 4 = “helped moderately” and 5 = “helped a great deal”.

#### Treatment integrity

In addition to evaluating participant ratings of the CBT-SF and HE conditions, treatment adherence and integrity assessment ensured that both intervention and control treatments were being delivered as intended. To ensure the highest level of treatment adherence, both interventions were manualised, with implementation monitored and therapists provided with ample training and supervision. One randomly selected audio recording per participant was rated by an independent assessor, who was a clinical psychologist and expert in CBT. Clinicians were given three ratings on a scale of one to eight: (1) *overall delivery* of the session, (2) *adherence* to the module being delivered and (3) *competency* in delivering the module. *Overall delivery* of the session in the CBT-SF condition was defined as the therapist’s adherence to the general CBT approach, including setting an agenda, presenting a rationale for therapeutic tasks, reviewing or assigning homework and maintaining the therapeutic relationship. In the HE control, the *overall delivery* of the session included an initial discussion of the participant's symptoms, presentation of educational information, discussion of the participant’s experiences of the specific topic and no use of individualised CBT-SF strategies. The independent assessor was instructed to determine whether clinicians avoided use of CBT-SF techniques and provide ratings accordingly. *Module adherence* and *competency* rated clinicians on how closely they followed key points set out in the treatment manuals and their ability to competently present this information in an informative and empathetic manner.

#### Feasibility

Finally, treatment completion and attrition rates were obtained for each group to determine treatment feasibility. Treatment completion indicated how many participants completed all eight CBT-SF or HE sessions, regardless of whether they completed follow-up appointments. Attrition rates refer to participants who withdrew from the study after completing treatment.

### Data analysis

Data were analysed using Jamovi version 2.3. Assumptions of normality and heteroscedasticity were assessed. Participant and treatment integrity variables were nonnormal with no transformations adequately providing correction. Therefore, these ratings were compared using Wilcoxon rank-sum tests with 95% confidence intervals. Completion and attrition rates were evaluated using chi-square tests of independence with 95% confidence intervals.

## Results

Sample demographic and injury characteristics are presented in Table 2. Table 3 displays medians, interquartile ranges and significance test results between CBT-SF and HE for participant ratings and integrity monitoring ratings. Wilcoxon rank-sum tests were conducted to evaluate whether there were any significant differences in face validity ratings (participant ratings of overall satisfaction, helpfulness for fatigue and helpfulness for sleep) and integrity monitoring ratings (module adherence, therapist competency and overall delivery). There were no significant CBT-SF vs. HE differences for participant satisfaction overall or in satisfaction for reducing fatigue symptoms specifically. However, the CBT-SF group rated helpfulness for sleep symptoms higher than those who received HE.

**Table 2:** Demographic and injury characteristics by treatment condition at baseline

	CBT-SF (*n* = 34) M (SD) or %	HE (*n* = 17) M (SD) or %	Range
Demographics			
Age at study entry	48.59 (14.82)	48.78 (11.95)	23–71
Sex (% male)	59%	65%	
Years of education	13.44 (1.76)	13.35 (2.51)	9–17
Injury characteristics			
Injury type (% TBI)	44%	41%	
Time since injury (months)	62.05 (62.32)	49.22 (53.23)	5–251
PTA duration (for TBI)	29.70 (35.10)^a^	14.60 (13.20)^b^	< 1–109
GCS score (for TBI)	7.5 (5.14)^c^	7.17 (4.36)^c^	3–15
Stroke mechanism (% ischaemic)	72%^c^	70%	
Stroke hemisphere (%)	Right (50%)^c^ Left (39%)^c^ Bilateral (11%)^c^	Right (30%) Left (40%) Bilateral (30%)	

*CBT-SF* Cognitive behavioural therapy for sleep disturbance and fatigue, *GCS* Glasgow Coma Scale, *HE* Health education, *M* mean, *PTA* Post-traumatic amnesia, *SD* Standard deviation, *TBI* Traumatic brain injury, ^a^Missing data from five participants, ^b^Missing data from two participants, ^c^Missing data from one participant

**Table 3:** Comparison of CBT-SF and HE participant ratings and integrity monitoring

	CBT-SF Median (IQR)	HE Median (IQR)	Wilcoxon test statistic
Participant ratings (1–5)			
Overall satisfaction rating	5 (1)	4 (1)	164
Fatigue helpfulness rating	4 (2)	3 (1.75)	245
Sleep helpfulness rating	4 (2)*	2.5 (1.75)*	103.50
Integrity monitoring ratings (1–8)			
Overall delivery of session	7 (1)	8 (1)	215
Module adherence	7 (1)*	8 (1)*	159.50
Competency in module delivery	8 (1)	8 (1)	200

^*^Significant at *p* < .001

On integrity monitoring ratings, CBT-SF and HE did not differ significantly in overall delivery or competency. The HE group had significantly higher module adherence compared to CBT-SF.

Lastly, chi-square tests of independence evaluated whether there were any significant differences in feasibility (completion rates and attrition rates) between HE and CBT-SF (Table 4). Results revealed no significant differences in completion or attrition rates between CBT-SF and HE.

**Table 4:** Comparison of CBT-SF and HE treatment characteristics

	CBT-SF %	HE %	*χ*^2^	Difference in %	95% *CI*
Treatment characteristics					
% Treatment completion rates	93.39%	87.50%	0.60	6.44%	4–17%
% Attrition rates	12.12%	6.25%	1.22	5.87%	3.6–8%

## Discussion

The first aim of this paper was to provide a detailed description and rationale for the HE design. A second aim was to evaluate face validity, treatment integrity and feasibility of HE in comparison with CBT-SF, to provide an indicator of treatment acceptability and suitability of HE as an active control. Overall, the hypothesis that there would be no significant differences in participant satisfaction/helpfulness ratings, treatment integrity or treatment feasibility was partially supported. While there were no differences in participants’ overall satisfaction or helpfulness ratings for reducing fatigue, the CBT-SF group had a significantly higher rating for sleep helpfulness compared to HE. Furthermore, treatment integrity monitoring ratings were not significantly different for overall delivery and therapist competency, but the HE group had significantly higher treatment adherence. Lastly, there were no differences in treatment completion or attrition rates. These results suggest that our HE active control condition is an adequate and appropriate comparator for CBT-SF in our active control pilot RCT.

Evaluation of post-treatment participant ratings indicated no differences in overall satisfaction or helpfulness in reducing fatigue between CBT-SF and HE groups. However, those who received CBT-SF reported significantly higher helpfulness for reducing sleep symptoms than those in the HE group. This most likely stems from the tailored and targeted sleep interventions implemented in the CBT-SF condition, which provided larger benefits than the generalised HE content. Indeed, preliminary analyses from the active control pilot RCT indicated that those who received CBT-SF had significantly greater improvement in sleep disturbance on the PSQI after treatment, compared to HE [8]. Furthermore, it is also possible that if participants were making self-directed lifestyle changes in response to the HE information, they may have perceived a larger impact on fatigue, such as engaging in regular exercise or eating a more balanced diet. It could be that these kinds of health-related changes may not translate as clearly to improvements in sleep symptoms, compared to individualised CBT-SF techniques.

Based on treatment integrity ratings, adherence to module components was significantly higher in the HE group compared to CBT-SF. This is not unexpected given the inherent flexibility of the CBT-SF intervention to adapt modules and strategies to participants' presenting problems. The HE condition, in contrast, had a predetermined set of educational material and generalised strategies to provide to participants, which was not to be tailored or modified to suit the participants’ specific needs. Despite the significant difference in this rating however, it is important to note that both HE and CBT-SF average ratings remained in the “very high” range for module adherence, and this difference was therefore unlikely to have significantly impacted treatment outcomes or participant ratings.

Limitations of our design must be acknowledged. First, we did not include a direct measure of treatment expectations prior to starting treatment. This would have been a useful addition to further evaluate the validity of our HE condition as an active control. Our analysis of post-treatment satisfaction ratings accounted for this limitation in part; however, future research may benefit from including a formal pretreatment measure. Secondly, as we were aware prior to conducting the RCT, the allocation of treatment group was not double blinded. Both therapists and participants knew the intervention in which they were engaging, with only follow-up research assistants blinded to condition allocation. This is nevertheless unavoidable for RCTs of psychological therapies. To minimise bias, both interventions were presented to participants as active treatments, so they would not have been aware that they were receiving a control condition.

Despite these limitations, we believe that the description and evaluation of our HE condition provides a valuable contribution to the current body of literature, and that our novel HE intervention was a feasible and acceptable active control group. To our knowledge, no other study has explicitly described the process of designing a HE control condition compared to a CBT intervention, and certainly not in individuals with ABI. Although often conducting high-quality RCTs, the development of non-specific control groups frequently goes undescribed, and in some cases, left out entirely, despite being a minimum requirement of reporting an RCT [31]. In fact, over half of published papers included in a review by Schroter and Glasziou et al. [32] did not provide treatment descriptions sufficient to allow replication, and for a third of treatments, the duration (intervals, frequency, length or timing) of intervention and control conditions was not clear. This makes it very difficult to compare between RCT trials who may both enlist a “health education” control condition but contain entirely different content, structure and monitoring. We hope that our description and evaluation can provide guidance for future RCTs employing HE and highlight the practical issues facing the development and evaluation of a novel control condition.

## Conclusion

Our ongoing active control pilot RCT aims to build on findings by Nguyen and Wong et al. [9, 10] by comparing the preliminary efficacy of CBT-SF with that of a HE condition, to account for non-specific effects of engaging in therapy [8]. As described above, we created a novel HE control condition in order to fulfil this requirement. We describe the rationale, considerations and specific design of this control condition, and evaluated participant ratings, integrity monitoring and treatment engagement. We believe that the HE control has adequate face validity, treatment fidelity and feasibility to be administered as an active control condition for comparison with CBT-SF. However, findings should be interpreted within the context of the study design, limited sample size and study population. In providing a real-world example of the practical and theoretical issues to consider in our design, we hope to provide a framework and guidance for future investigators wishing to implement a control condition of a similar nature.

## Data Availability

The datasets generated and analysed during the current study are not publicly available due to continued data collection and recruitment but are available from the corresponding author on reasonable request.

## Abbreviations

RCT: Randomised controlled trial
CBT: Cognitive behavioural therapy
CBT-SF: Cognitive behavioural therapy for sleep disturbance and fatigue
HE: Health education
ABI: Acquired brain injury
TBI: Traumatic brain injury
